# Real-Time Placental Perfusion on Contrast-Enhanced Ultrasound and Parametric Imaging Analysis in Rats at Different Gestation Time and Different Portions of Placenta

**DOI:** 10.1371/journal.pone.0058986

**Published:** 2013-04-01

**Authors:** Yi-Jie Zhou, Man-Li Yuan, Rui Li, Li-Ping Zhu, Zhao-Hui Chen

**Affiliations:** Department of Ultrasound, Southwest Hospital Affiliated to Third Military Medical University, Chongqing, China; Institut Jacques Monod – UMR 7592 CNRS – Université Paris Diderot, France

## Abstract

**Objectives:**

To quantitatively analyze placental perfusion in a rat model at different gestation time and different portions of placenta by real-time contrast-enhanced ultrasound (CEUS) and parametric imaging analysis.

**Materials and Methods:**

Sixty pregnant rats at different gestation time (15 dys,17 days and 20 days) were injected intravenously with microbubbles (5×10^5^ microbubbles /ml, 1.0 ml/kg), and cadence contrast pulse sequencing (transmission frequency of 7 MHz, mechanical index 0.18) was performed. Dynamic enhancement changes in placenta at different gestation time and different portions of placenta were measured and enhancement parameters analyzed with software. Correlation between enhancement parameters and average area densities of placenta vascular compartment was compared.

**Results:**

The pattern and real-time sequence of enhancement in uterus and placenta were clearly depicted by CEUS. The time-to-peak enhancement was earlier in central portion than that in peripheral portion (12.30±6.33s vs 36.26±10.65 s, *p* = 0.005), and peak intensity of enhancement is much higher in central portion than that in peripheral portion (30.20±2.85 dB vs 20.95±6.25 dB, *p* = 0.000). The peak intensity of enhancement at day 15 (27.70±4.47 dB) was lower than that at day 17 (30.20±2.85 dB, *p* = 0.042) and at day 20 (31.85±4.41 dB, *p* = 0.015) of gestation. Significant correlation between average area densities of vascular compartment and the peak intensity of enhancement was identified in placenta at different gestation time (*p*<0.05). The average area densities of vascular compartment was higher in central portion than that in peripheral portion and has significant correlation with peak intensity of enhancement of the two potions (*p*<0.01).

**Conclusion:**

CEUS is feasible to depict real-time sequence and quantitative parameters of perfusion in different portion of placenta at different gestational time in a rat model.

## Introduction

The placenta represents the interface where nearly all exchanges of respiratory gasses, nutrients and waste between the mother and fetus. Trans-placental exchange depends primarily on the rate of maternal placental and fetal blood flow [1]. Failure of the trophoblast invasion of the maternal spiral arteries and reduced utero-placental perfusion are associated with fetal growth restriction and preeclampsia [2]–[4]. A safe and accurate means of quantification of placental perfusion would allow enhanced investigation of the pathogenesis and severity of fetal growth restriction, preeclampsia, and estimation of the response of vasodilatory and antithrombotic treatments [5], [6]. However, the study of placental patho-physiology and perfusion in vivo has been yet a challenging work.

Doppler ultrasonography is a noninvasive and widely available imaging method [7]. However, Doppler – derived numeric indices do not reliably correlate with clinical disorders known to be associated with reduced utero-placental perfusion, and has limited value in prediction of preeclampsia, intrauterine growth restriction, and perinatal death [8], [9]. Placental perfusion has been investigated in many ways, including the radioactive microspheres and angiography [10], [11] but concerns for fetal and mother radiation exposure place severe restrictions for human applications. Placental perfusion has been studied by magnetic resonance imaging with contrast agents [12], [13]. There are several papers evaluating perfusion/permeability in rats/mice models using MRI in normal or pathological conditions in recent years. These studies have demonstrated that placental perfusion/permeability can be measured in vivo in normal or pathological conditions using MRI with different imaging sequence or different contrast agent [14], [15], [16], [17]. It is interesting to compare MRI and contrast enhanced ultrasound (CEUS) in this area, but this is beyond the scope of our present study, and comparison between the two imaging modalities may be conducted in future work. Nevertheless, MRI may have some disadvantages: (1) high cost and low availability; (2) paramagnetic agents may cross the placenta into the fetal circulation shortly after maternal administration, and the impact on safety of fetus is unknown; (3) additionally, the fetus was exposed to strong magnetic fields which might be deleterious for fetal heart and audition [18].

CEUS has been used in clinics, especially in evaluating the blood perfusion of organs such as heart, liver, brain and kidneys [19], [20], [21], [22]. As the size of microbubbles (with diameter of about 2–8 μm) is similar to that of red blood cells and they are purely intravascular and do not infuse into the interstitium of tissue, therefore, microbubbles can be used as blood tracers [22]. However, there is little knowledge about placental perfusion in vivo yielded from real-time grey scale CEUS [23]. It is demanding to seek investigative technique which is safe, accurate and widely available to study placental pathophysiology and perfusion in vivo. The purpose of our study was to quantitatively investigate placental perfusion by using real-time CEUS and parametric imaging analysis in a rat model at different gestation time and different portions of placenta.

## Materials and Methods

### Animal preparation and contrast agent

Sprague Dawley (SD) rats weighting 245.1 g −380.3 g (mean 285.8 g) were mated at the supplier laboratory (the experimental animal center of Third Military Medical University). Pregnant rats were divided into five groups according to different gestation times (at 8 days, 10 days, 15 days, 17 days and 20 days of gestation). This study was carried out in strict accordance with the recommendations in the Guide for the Care and Use of Laboratory Animals of Third Military Medical University. All surgery was performed under sodium pentobarbital anesthesia, and all efforts were made to minimize suffering of experimental animals.

SonoVue (Bracco Imaging B.V, Geneva Switzerland), a phospholipid microbubbles encapsulating SF6, was applied as the ultrasound contrast agent. Five ml physiologic saline was injected into the bottle with the agent powder inside, and the bottle was shaken to form a microbubble suspension (5×10^5^ microbubbles /ml) according to the instructions of manufacture. The average diameter of the microbubbles was 2.5 μm [24].

### Experimental procedures

After anesthesia by intraperitoneal injection of 1% pentobarbital sodium (40 mg kg^−1^), the fur of rat abdomen was depilated with electric shaver as the acoustic window for ultrasound imaging. Ultrasound examinations were performed with an Acuson Sequoia 512 ultrasound unit (Simens Medical Solutions, Santa Clara, Calif) with a 15L8W linear transducer. Initially, the pregnant rats were examined in conventional B-mode ultrasound (with transmission frequency of 14 MHz, mechanical index of 0.51, depth of 2.5 cm) for the imaging of the fetus and placenta, according to which, their location was marked on abdominal surface. The placenta was shown longitudinally with the largest dimensions, and then the transducer was fixed in this position. Only one investigator (Rui Li) was assigned to select placenta image plane and fix transducer to mitigate image variation. One placenta near the posterior wall of uterine was chosen in each rat for CEUS imaging to reduce the reverberation artifacts near the transducer. This is feasible because there were several gestational sacs in one uterine tube. Ultrasound device was set in cadence contrast pulse sequencing (CPS) mode (with transmission frequency of 7 MHz, mechanical index 0.18). Then, a bolus of prepared SonoVue microbubbles (1.0 ml/kg) were injected through caudal vein followed by 1 ml saline to wash the tube according to our previous experiment [25)]. while simultaneously digital recording of 120-second cine loops with a frame rate of 8 frames/second. After ultrasound examination, the rat was killed, rat belly was cut at the marked site, and the placenta was resected. The placenta was fixed in 10% formaldehyde, and routinely processed. Specimens were cut parallel to the long axis of placenta into sections of 5-μm in thickness, and stained with hematoxylin and eosin. Sections of the placenta were examined under light microscopy (BX51, Olympus, Japan). For quantification of vascular compartment, A 1000 optical magnification was used and 10 random fields of view of on a section were taken for imaging analysis using a Image Pro Plus software tool (Media Cybernetics Inc, USA). Average area densities of vascular compartment were calculated.

### Parametric analysis

The cine loop Of CEUS was analyzed with in-house ACQ software (Axius^TM^ Auto Tracking Contrast Quantification, Simens Medical Solutions). Regions of interest (ROI) was drawn by one investigator (Man-Li Yuan) to mitigate variation in this study. The placenta was shown longitudinally with the largest dimensions. It is not appropriate to use a fixed ROI size and placement because the shape of contrast enhanced placenta is irregular and the size varied. ROI were drawn manually around the contours of the lateral wall of uterine, central portion (about 1/3 of the enhanced placenta in thickness at the central part) and peripheral portion (about 1/3 of the enhanced placenta in thickness at the peripheral part) of enhanced placenta respectively on the image that visually seemed to have the largest area of obvious enhancement on CEUS. Afterwards, time-intensity curves (TICs), arrival time (AT) of microbubbles, time-to-peak intensity (TTP) and peak intensity of enhancement (PI) were calculated automatically by the software. Intraobserver variability of PI of enhanced placenta at15 days and 17 days of gestation was tested for the reason that PI has significant correlation with area densities of vascular compartment of placenta. Every placenta was measured at four different visits by the same investigator (Man-Li Yuan) from the cine loops after CEUS in 10 randomly selected rats at 15 days and 17 days of gestation respectively. There was a one week delay between each visit and the investigator was masked to the previous results.

For quantification of average area densities of vascular compartment, 10 random fields of view on a section with 1000 optical magnification were taken for imaging analysis using Image Pro Plus software tool. The area of vascular compartment was drawn manually and then marked red automatically by Pro Plus software tool. The following equation was used to determine average surface areas of vascular compartment: average area densities of vascular compartment  =  total surface area of vascular compartment/total surface area of 10 random fields of view on a section of one placenta, and the results were calculated automatically by Pro Plus software tool.

### Statistical analysis

The data are presented as mean ± SD (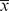
±SD). For statistical comparison between groups at different gestation time, analysis of variance (ANOVA) was performed, A *p-*value less than 0.05 was considered statistically significant. Correlation between CEUS and histological parameters of placenta was tested by Spearman bivariate correlate analysis, and with a *p-*value less than 0.05 indicating significant correlation. Intraclass correlation coefficients (ICCs) was calculated to test intraobserver variability [26] of peak intensity of enhancement measurement. Comparison was made of the differences between the four measurement results obtained by the same observer. ICC can be grouped with values less than 0.20 implying poor correlation, 0.21–0.40 fair, 0.41–0.60 moderate, 0.61–0.80 good and 0.81–1.00 very good correlation [27].

## Results

### Real-time CEUS of placenta

**Figure 1:**
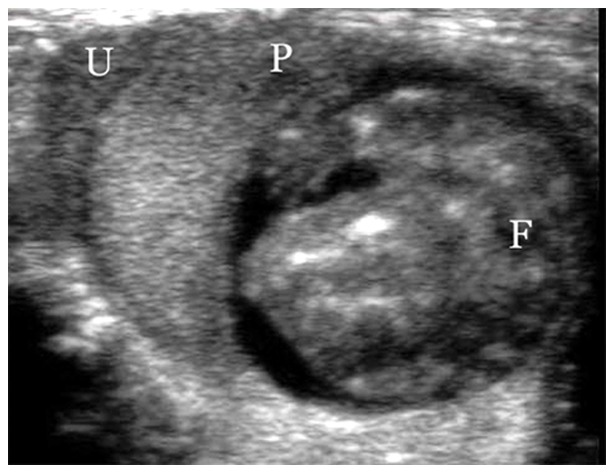
The anatomic structure of uterus (U), placenta (P) and fetus (F) were clearly depicted at 15 day of gestation by conventional ultrasound with 15L8W linear transducer.

**Figure 2:**
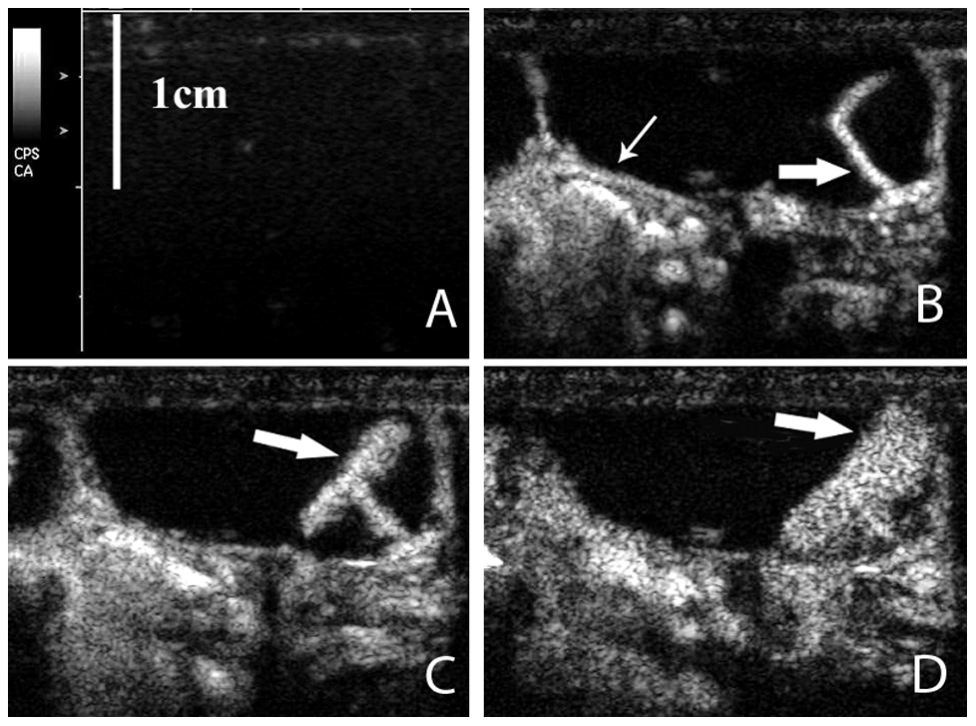
Typical pattern and sequence of placental enhancement after bolus microbubble injection on contrast-enhanced ultrasound (CEUS) with cadence contrast pulse sequencing (CPS) mode (with transmission frequency of 7 MHz, mechanical index <0.2). Before injection, the uterus and placenta could not be visualized (image A). The uterus (small arrow) and central arterial canal (large arrow) were enhanced 4 seconds after microbubble injection (image B). The central potion (→) of placenta was enhanced quickly afterword (image C, 11 seconds after injection). Thereafter, the maternal blood flow percolate back to peripheral portion of maternal side, and the entire placenta (→) was enhanced gradually (image D, 45 seconds after injection).

Although the uterine tubes, gestational sac were differentiated at day 8 in pregnant rat by conventional ultrasound with 15L8W linear transducer, the anatomic structure of fetus and placenta were not clearly distinguished until 15 days of gestation (Fig. 1). Therefore, CEUS was performed at day 15, day 17 and day 20 of gestation respectively with 20 rats in each gestation time group. Under CPS contrast mode, The uterine was enhanced first (about 0.5 second after injection of microbubbles), then the central arterial canal and its branches enhanced quickly and fetal side of labyrinth enhanced afterward (about 1.5 seconds after injection of microbubbles), and microbubbles then percolated back through the intervillous space of the labyrinth to the maternal side, leading to enhancement of the entire placenta (Fig. 2). No fetal enhancement was observed in gestational sac of all pregnant rats.

### Parametric imaging analysis

**Figure 3:**
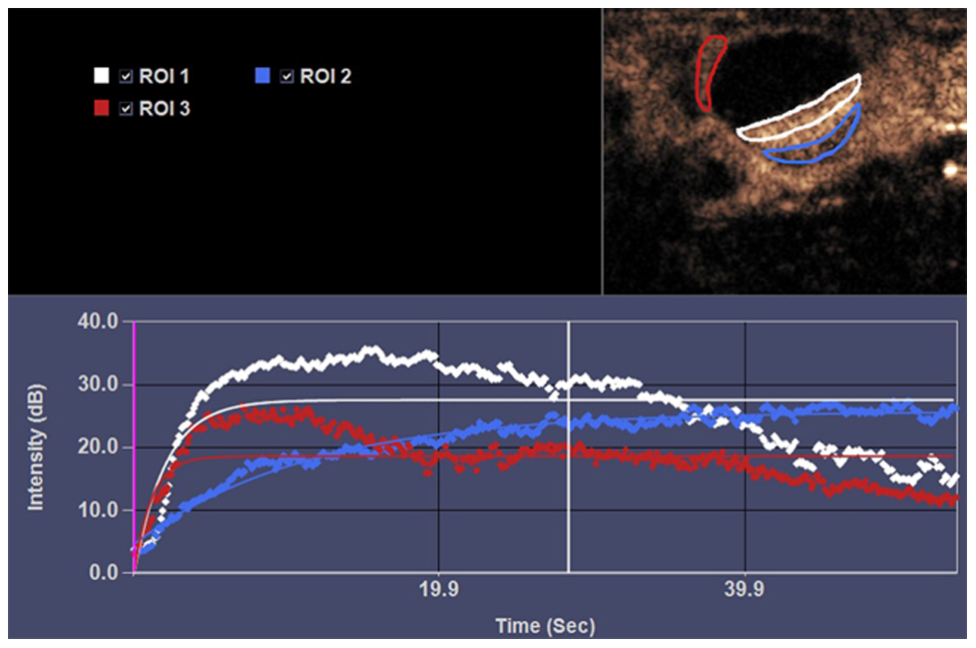
Examples of time-intensity curves of enhancement obtained in a pregnant rat at day 17 of gestation. Regions of interest (ROI) were drawn around the contours of the lateral wall of uterine (red circle), central portion (white circle) and peripheral portion (blue circle) of the placenta respectively on the image that visually seemed to have the largest area of obvious enhancement on CEUS. The central potion shows a faster and higher enhancement pattern (white line) than that of peripheral portion (blue line) of placenta.

Parametric imaging analysis was failed in 5 rats due to accidental movement of the rat during real -time CEUS image acquisition, and the failure rate was 8.3% (5/60). We repeated the CEUS imaging with other rats of the same gestation time for we had prepared 25 -28 rats of each gestation time group (15 day, 17 day and 20 day) before real- time CEUS start. The arrival time of microbubbles (0.48±0.04 s vs 1.55±0.69 s, *p*<0.01) and time-to-peak enhancement (4.98±0.71 s vs 13.63±1.08 s, *p*<0.01) were faster in uterine than that in placenta, and there was no difference in peak intensity of enhancement between uterine and placenta (27.36±4.95 s vs 27.70±4.47 s, *p*>0.05). When the regions of interest (ROI) were drawn in the central portion (corresponding to labyrinth) and peripheral portion (corresponding to decidua basalis and metrial triangle) of placenta respectively at day 17 of gestation (Fig. 3), the arrival time of microbubbles and time-to-peak enhancement were earlier in central portion than that in peripheral portion (*p* = 0.005, *p* = 0.000, respectively), and peak intensity of enhancement is much higher in central portion than that in peripheral portion of placenta (*p* = 0.000, Table 1). The peak intensity of enhancement of central potion of placenta (corresponding to labyrinth) at day 15 was lower than that at day 17 (*p* = 0.042) and at day 20 (*p* = 0.015) of gestation. There was no statistical difference between 17 days and 20 days of gestation in peak intensity of enhancement of placenta (*p* = 0.664). The arrival time of microbubbles and time-to-peak enhancement were similar among the day 15, day 17,and day 20 groups (*p*>0.05, Table 2).

**Table 1:** Perfusion parameters of CEUS in different portions of placenta at day 17 of gestation.

	AT (s)	TTP (s)	PI (dB)
Central portion (n = 20)	1.29±0.69	12.30±6.33	30.20±2.85
Peripheral portion (n = 20)	2.79±1.77^▴^	36.26±10.65^▴^	20.95±6.25^▴^

Note: AT: arrival time of microbubbles; TTP: time-to-peak of enhancement;

PI: peak intensity of enhancement; ^▴^: *p*<0.01 as compared with central portion of placenta.

**Table 2:** Perfusion parameters of CEUS in central potion of placenta at different gestation time.

Gestation time (d)	AT(s)	TTP(s)	PI(dB)
15(n = 20)	1.54±0.91	13.63±7.08	27.70±4.47
17(n = 20)	1.29±0.69	12.30±6.33	30.20±2.85^▴^
20(n = 20)	1.26±0.61	12.02±5.42	31.85±4.41^▴^

Note: AT: arrival time of microbubbles; TTP: time-to-peak of enhancement;

PI: peak intensity of enhancement.^ ▴^: *p*<0.05 as compared with day 15 of gestation.

### Intraobserver measurement reproducibility

Intraobserver ICCs were high for PI of placenta enhancement (0.97 in gestation day 15 group and 0.96 in gestation day 17 group), indicating good intraobserver measurement reproducibility. Results of four different PI measurements of placenta enhancement at 15 day and 17 day were shown in supplementary materials (Table S1, Table S2).

### Histology and measurement of vascular densities

**Figure 4:**
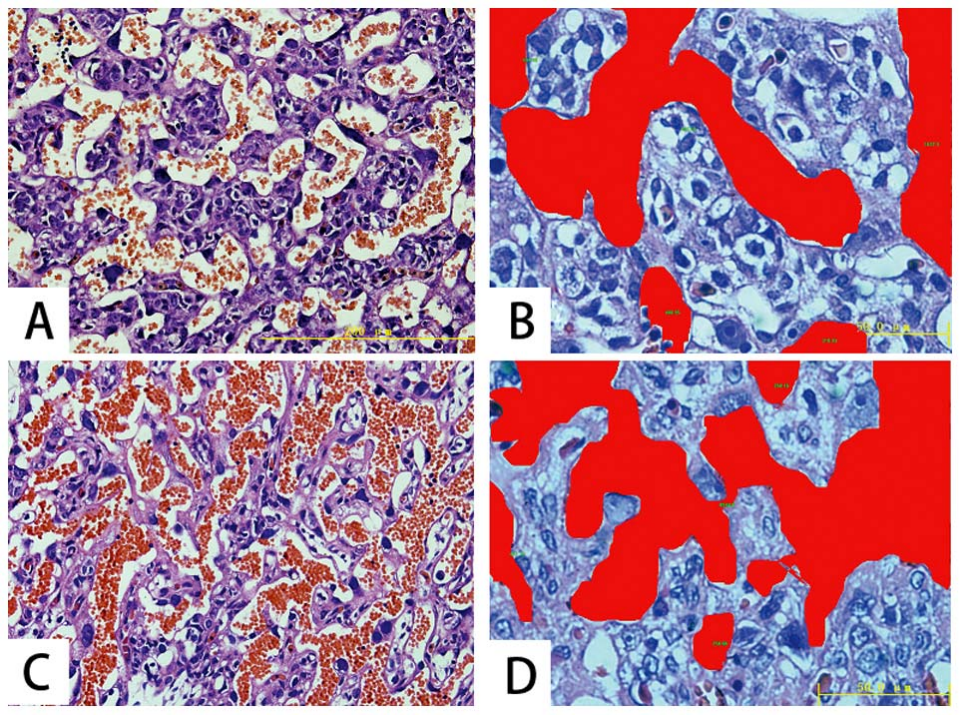
Example of histological analysis of area densities of maternal vascular compartment corresponding to the labyrinth on placental tissue section. Image A shows the histological structure within the labyrinth at day 15 of gestation. Mononuclear trophoblast cells that lines maternal blood sinusoids which was filled with maternal red blood cell.(image A, H&E-stained ×400). The area of maternal vascular compartment was drawn manually and marked red (image B, D ×1000) automatically by Pro Plus software tool. The area density of maternal vascular compartment was increased at day 17 (image D) than that at day 15 (image B) of gestation.

Histologically, the average area densities of maternal vascular compartment (Fig. 4) was increased significantly from 15 days to 17 days of gestation (*p* = 0.00), but without obvious increase from 17 days to 20 days (*p* = 0.676, Table 3). Significant correlation between average area densities of maternal vascular compartment and the peak intensity of enhancement of placenta was identified in the three groups of different gestation time (Table 3). The average area densities of maternal vascular compartment was higher in central portion than that in peripheral portion of placenta and has significant correlation with peak intensity of enhancement of the two potions (Table 4).

**Table 3:** Correlation between PI and average area density of maternal vascular compartment in central potion of placenta at different gestation time.

Gestation time (d)	PI	Vascular area density	*r*	*p*
15(n = 20)	27.70±4.47	0.34±0.04	0.449	0.025
17(n = 20)	30.20±2.85	0.49±0.07	0.841	0.000
20(n = 20)	30.85±4.41	0.51±0.07	0.859	0.000

Note: PI: peak intensity of enhancement; vascular area density: average area density of maternal vascular compartment.

**Table 4:** Correlation between PI and average area density of maternal vascular compartment at different portion of placenta at 17 day of gestation.

	PI	Vascular area density	*r*	*p*
Central portion	30.20±2.85	0.49±0.07	0.841	0.000
Peripheral portion	20.96±6.25	0.10±0.05	0.944	0.000

Note: PI: peak intensity of enhancement; vascular area density: average area density of maternal vascular compartment.

## Discussion

Placental perfusion is crucial for normal fetal development and growth, but evaluation of placental perfusion in vivo has been yet a challenging task. Investigator have used ultrasound contrast agents to examine utero-placental perfusion with pulsed-wave and color Doppler ultrasound [28], [29]. Although Doppler signals could be enhanced significantly, the authors can not evaluate real-time quantitative changes in contrast effects because rapid destruction of microbubbles by high mechanical index Doppler ultrasound. In addition, ultrasonographic picture will be blurred by Doppler “blooming” artifact [30]. Ragavendra and Tarantal used contrast agent Aerosomes and grey scale harmonic imaging technique to investigate utero-placental perfusion in the third trimester in three rhesus monkeys. Due to superior spatial resolution afforded by grey scale imaging enables better and more accurate delineation of echo-contrast filled blood vessels than with color or power Doppler imaging, and they demonstrated placental intervillous blood flow without visualization of fetal blood circulation within the chorionic villi [31]. Barth and colleagues have recorded time-intensity curves depicting the temporal nature of contrast agent-induced changes in the ultrasound appearance of intervillous blood flow in nine pregnant baboons, and demonstrated the feasibility of constructing such curves from intervillous space with intravascular contrast agent and computer assisted video densitometry [23].

Our study differs from the above reports in the following aspects: (1) we were able to study blood perfusion in a much smaller rat placenta using high frequency linear transducer with high spatial resolution and CPS contrast mode at low mechanical index that allows detecting very small amounts of contrast agent with excellent tissue suppression [32]. The advantages of pregnant rat model are including much lower cost, easily available, practicality in larger number of animals which is inevitable for statistical analysis of perfusion parameters, and research is not restricted by the inherent ethical and practical limitations associated with the human.;(2) Our method allows one to place the sample volume for obtaining contrast intensity data exactly over a small region of interest guided by direct visualization within the high resolution ultrasound images. Relying on the feature, we successfully obtained the time-intensity curves and related parameters of contrast enhancement of uterus, central portion and peripheral potion of the placenta respectively in pregnant rats ; (3) placental perfusion was compared between different time of gestation and different portions, (4) Correlation between ultrasound contrast enhancement parameter and histological average area densities of vascular compartment was investigated.

In our pregnant rat model, although the uterine tubes, gestational sac and embryo were differentiated at day 8 by conventional ultrasound with linear transducer at frequency of 14MHz, the anatomic structure of fetus and placenta were not clearly distinguished until 15 days of gestation. Therefore, real-time CEUS was successfully performed at day 15, day 17 and day 20 of gestation in matured rat placenta in our study to demonstrate the utero-placental perfusion of maternal blood.

We were able to depict clearly the temporal enhancement sequence of uterine, central arterial canal, central portion of the labyrinth and finally, microbubbles bounced slowly back through the intervillous space of the labyrinth to the peripheral portion (maternal side) of placenta due to superior spatial resolution afforded by high frequency ultrasound (7 MHz) and high temporal resolution (8 frames/second) of CPS contrast imaging. Our findings are in keeping with those of Adamson et al, who depicted placental circulation on the basis of vascular casts and histological studies that after passing through the arterial canals, maternal blood then percolates back to the apical (maternal) side of the placenta through tortuous, anastomosing, trophoblast-lined sinusoids in the labyrinth. The sinusoids coalesce into larger channels that traverse the spongiotrophoblast and giant cell layers and lead into maternal endothelial cell-lined, large venous sinuses in the decidua basalis. The fetal and maternal blood then travel through the microcirculation within the labyrinth in opposite directions [33].

Our quantitative investigations showed that the arrival time of microbubbles and time-to-peak enhancement were earlier in central portion (corresponding to labyrinth) than that of peripheral potion in maternal side (corresponding to decidua basalis and metrial triangle) of the placenta, and peak intensity of enhancement is much higher in central portion than peripheral potion in maternal side of the placenta. These results illustrated in vivo that oxygen-rich maternal blood is delivered via the central canals directly to the base (central portion) of the labyrinth, showing a quick and high perfusion feature in central portion compared to a slower and lower perfusion feature in peripheral potion in maternal side of the placenta. Previous placental perfusion MRI imaging with contrast agents in a mouse model also showed that maximum of enhancement was reached in center of placenta corresponding to labyrinth region [13]. Our histological analysis showed that average area densities of vascular compartment was higher in central portion than that in peripheral portion of placenta and has significant correlation with peak intensity of enhancement of the two areas, demonstrating that more microbubbles were accumulated in relatively larger vascular compartment area in central portion than that in peripheral portion when microbubble concentration up to the achievement of a plateau phase. Some investigations have demonstrated that numeric indices derived from contrast ultrasound in vivo correlate well with more standard techniques employing electromagnetic flow probes or radioactive microspheres [34], [35]. Tissue perfusion quantification by CEUS analysis has been accepted in clinical as well as in animal models recently [36]. Determination of the degree of tissue contrast enhancement relies on the accurate distinction between microbubble backscatter signals and tissue background. The cadence contrast pulse sequencing (CPS) mode applied in our study works by interrogating each imaging line a number of times with pulses with various amplitude and phases. Both harmonic and non-linear fundamental signals are represented on a grey-scale map suppressing the linear fundamental echoes from native tissues that allows detecting very small amounts of contrast agent with excellent tissue suppression [32], [36]. Analysis of time-intensity curves from CPS contrast imaging revealed early quantitative vascular changes in antiangiogenic treatment response in metastatic cancer, and the results from CPS contrast imaging were in good agreement with dynamic contrast-enhanced MRI [37].

Histological analysis of this study demonstrated that, the average area densities of maternal vascular compartment was increased significantly from 15 days to 17 days of gestation, but with no remarkable increase from 17 days to 20 days. This results is similar to a previous study by Coan et al [38] using histological stereology, who found that the absolute volume and surface area of mouse maternal blood spaces (MBS) expand rapidly between day14.5 and day16.5 of gestation, with no increase thereafter. Our CEUS investigation revealed that the peak intensity of enhancement of placenta at day 15 was lower than that at day 17 and at day 20 of gestation. There was no statistical difference between 17 days and 20 days of gestation in peak intensity of enhancement of placenta. Significant correlation between average area densities of maternal vascular compartment and the peak intensity of enhancement of placenta was identified in the above mentioned three groups at different gestation time. These experimental results indicate that CEUS with CPS imaging mode may reflect quantitative changes of maternal vascular compartment in vivo during different stages of rat pregnancy.

This study has some limitations: (1) subjective choice of region of interest, which may offer part of the variability of enhancement measurements; (2) precision might be hindered by section thickness of ultrasound probe, which induced some volume averaging. However, this limitation also exist in other dynamic imaging modality such as placental perfusion MRI imaging [13]. In this primary study, though we try to fix transducer to show rat placenta longitudinally with the largest dimensions on CEUS, and only one investigator was assigned to select placenta image plane and fix transducer to mitigate image variation. Ultrasound transducer provided information in only a single slice of placenta tissue. Therefore, it is difficult to image exact the same plane from one observer to another. Newly emerged three-dimensional CEUS perfusion imaging may allow a significant reduction in this error caused by slice selection and improves reproducibility of perfusion measurement of animal kidneys [39], [40]. Whether three-dimensional perfusion imaging may significantly improve repeatability of quantitative parametric analysis of placental perfusion in rats remains open to future work; (3) the relationship between enhanced signal intensity and concentration of microbubbles should be determined before experiment to select suitable dosage for intravenous injection; (4) concerns of the use of ultrasound contrast agent during pregnancy. However, there is increasing evidence that CEUS is safe during pregnancy [29], [41], [42]. In addition, microbubbles do not cross the placenta and diagnostic CEUS with microbubbles will not increase the permeability of placenta [25].

In summary, we demonstrated a technique of real-time CEUS for quantitatively dynamic placental perfusion study in a rat model. The technique demonstrated high temporal and spatial resolution and can reveal real-time sequence and quantitative parameters of perfusion in different portion of placenta and uterus at different gestational time. Therefore, our study may provide the basis for future in vivo studies of placental perfusion related disorders, such as fetal growth restriction, preeclampsia, intrauterine death and estimation of the response of vasodilatory and antithrombotic treatments of placenta in rat models of human diseases.

## Supporting Information

Table S1 **Results of four different measurements of peak intensity (PI) of placenta enhancement at 15 day of gestation in 10 rats.**
(DOC)

Table S2 **Results of four different measurements of peak intensity (PI) of placenta enhancement at 17 day of gestation in 10 rats.**
(DOC)
