# Supplementary material for: Real-Time Placental Perfusion on Contrast-Enhanced Ultrasound and Parametric Imaging Analysis in Rats at Different Gestation Time and Different Portions of Placenta
Source: PLoS One. 2013 Apr 1;8(4):e58986. doi: 10.1371/journal.pone.0058986 (PMC3613345; doi:10.1371/journal.pone.0058986)
Supplement: Table S1 — Results of four different measurements of peak intensity (PI) of placenta enhancement at 15 day of gestation in 10 rats. (DOC) [file pone.0058986.s001.doc]

**Table S1.** Results of four different measurements of peak intensity (PI) of placenta enhancement at 15 day of gestation in 10 rats

| No. of rats | 1^st^ measurement | 2^nd^ measurement | 3^rd^ measurement | 4^th^ measurement |
| --- | --- | --- | --- | --- |
| 1 | 25.21 | 25.53 | 25.38 | 25.41 |
| 2 | 22.17 | 22.58 | 22.47 | 23.34 |
| 3 | 34.75 | 33.52 | 34.66 | 33.82 |
| 4 | 33.35 | 33.28 | 33.11 | 33.57 |
| 5 | 22.10 | 22.36 | 22.00 | 22.65 |
| 6 | 27.32 | 27.01 | 27.35 | 27.81 |
| 7 | 23.59 | 23.31 | 23.59 | 23.62 |
| 8 | 33.76 | 34.27 | 33.92 | 33.43 |
| 9 | 24.17 | 24.21 | 24.42 | 23.87 |
| 10 | 31.67 | 31.40 | 31.62 | 31.46 |
