# Supplementary material for: Real-Time Placental Perfusion on Contrast-Enhanced Ultrasound and Parametric Imaging Analysis in Rats at Different Gestation Time and Different Portions of Placenta
Source: PLoS One. 2013 Apr 1;8(4):e58986. doi: 10.1371/journal.pone.0058986 (PMC3613345; doi:10.1371/journal.pone.0058986)
Supplement: Table S2 — Results of four different measurements of peak intensity (PI) of placenta enhancement at 17 day of gestation in 10 rats. (DOC) [file pone.0058986.s002.doc]

**Table S2.** Results of four different measurements of peak intensity (PI) of placenta enhancement at 17 day of gestation in 10 rats

| No. of rats | 1^st^ measurement | 2^nd^ measurement | 3^rd^ measurement | 4^th^ measurement |
| --- | --- | --- | --- | --- |
| 1 | 29.01 | 28.92 | 29.58 | 28.92 |
| 2 | 27.90 | 27.15 | 27.96 | 27.22 |
| 3 | 30.31 | 31.15 | 30.03 | 30.12 |
| 4 | 33.22 | 33.19 | 32.81 | 32.93 |
| 5 | 33.41 | 33.23 | 32.97 | 33.13 |
| 6 | 33.84 | 33.73 | 33.92 | 32.86 |
| 7 | 32.90 | 32.76 | 32.61 | 32.71 |
| 8 | 33.61 | 33.84 | 33.06 | 33.01 |
| 9 | 25.30 | 25.43 | 25.25 | 26.79 |
| 10 | 26.80 | 26.92 | 26.78 | 26.45 |

Estimation of intraclass correlation

(Maximum likelihood estimator, common correlation model)

$$\begin{matrix} X_{i}\sim N(\mu_{i},\sum_{i}) \\ \mu_{i}=(\mu,\mu,\cdots,\mu)_{1\times n_{i}} \\ \sum_{i}=\left[\begin{matrix} \begin{matrix} \sigma^{2} & \rho\sigma^{2} & \cdots& \rho\sigma^{2} \end{matrix} \\ \begin{matrix} \rho\sigma^{2} & \sigma^{2} & \cdots& \rho\sigma^{2} \end{matrix} \\ \begin{matrix} & \cdots& & \cdots\end{matrix} \\ \begin{matrix} \rho\sigma^{2} & \rho\sigma^{2} & \cdots& \sigma^{2} \end{matrix} \end{matrix}\right]_{n_{i}\times n_{i}} \\ \end{matrix}$$

$$n_{i}=n=4$$

$$r_{P}=\frac{1}{kn(n-1)S_{x}^{2}}\mathop{\sum_{i=1}^{k} \sum_{j=1}^{n} \sum_{l=1}^{n} (x_{ij}}\limits_{j\neq l} -\bar{x})(x_{il}-\bar{x})$$

$\bar{x}=\frac{1}{nk}\sum_{i=1}^{k} \sum_{j=1}^{n} x_{ij}$,$S_{x}^{2}=\frac{1}{nk-1}\sum_{i=1}^{k} \sum_{j=1}^{n} (x_{ij}-\bar{x})^{2}$

From the data in table 1:$k=10$,$n=4$,$\bar{X}=27.8265,S_{x}^{2}=22.284$,$r_{P}=\frac{2\times1297.09}{10\times4\times3\times22.284}=0.970121$

From the data in table 2 : $k=10$,$n=4$,$\bar{X}=30.5433,S_{x}^{2}=8.7787$, $r_{P}=\frac{2\times503.7879}{10\times4\times3\times8.7787}=0.9564588$
